# Incidentally Detected Solitary Metastatic Melanoma of the Spleen Without Known Primary: A Case Report

**DOI:** 10.7759/cureus.38530

**Published:** 2023-05-04

**Authors:** Sumukh Arun Kumar, Chidambaram Ramasamy, Masood Pasha Syed, Archana Agarwal

**Affiliations:** 1 Internal Medicine, Saint Vincent Hospital, Worcester, USA; 2 Hematology and Oncology, University of Pittsburgh Medical Center, Pittsburgh, USA; 3 Hematology and Oncology, Saint Vincent Hospital, Worcester, USA

**Keywords:** skin cancer, metastatic melanoma, splenic mass, melanoma, splenic melanoma

## Abstract

Splenic masses could be secondary to infection or due to benign and malignant cancers. Due to its anatomy and microenvironment, the spleen is relatively protected from cancer spread. However, melanomas are one of the few cancers that metastasize to the spleen, but only 2% of these metastasize as solitary splenic masses. Among such a small fraction, only a handful have been reported without a known primary.

Our patient, an elderly male in his early 60s, was diagnosed with metastatic melanoma of the spleen following a biopsy of the incidentally detected isolated splenic mass. Complete ocular, oral, and dermatological inspections were unremarkable for a probable primary. He responded well to immunotherapy and total splenectomy with no recurrence.

Due to advanced imaging modalities in the modern era, the probability of isolated splenic masses as an initial presentation will increase, and a high index of clinical suspicion should be maintained for metastatic cancer as one of the differentials.

## Introduction

Splenic masses could range from infectious causes such as abscess to noninfectious etiology such as lymphoma, hamartoma, or angiosarcoma [1]. Fluorodeoxyglucose (FDG)-avid splenic tissue reflects metabolically hyperactive tissue such as abscesses, lymphoma, or solid tumor metastasis. Metastasis to the spleen is relatively uncommon, and even when it is invaded, it is usually a presentation of multi-visceral disseminated cancer [2]. Very few cases of an isolated splenic mass as an initial presentation of metastatic melanoma have been reported [3].

## Case presentation

An elderly male in his early 60s with a significant medical history of type 2 diabetes mellitus and hypertension and a 50-pack-year smoking history was evaluated in the clinic for an increase in the size of isolated right upper lobe solid pulmonary nodule on routine low-dose computed tomography (CT) of the chest surveillance. His pulmonary nodule increased from 6 mm to 9 mm over 12 months. He endorsed unintentional weight loss of 18 lbs over the same time. He denied any respiratory symptoms during the visit. He quit smoking 10 years ago. His family history was significant for colon cancer in a sibling at the age of 56. He has no previous history of any malignancy. He had a normal routine colonoscopy four weeks prior. His vitals, physical examination, and basic laboratory results, including lactate dehydrogenase (LDH), were unremarkable. However, due to the nodule’s growth and location and his significant smoking history, the decision was made to further evaluate with positron emission tomography-computed tomography (PET-CT).

**Figure 1:**
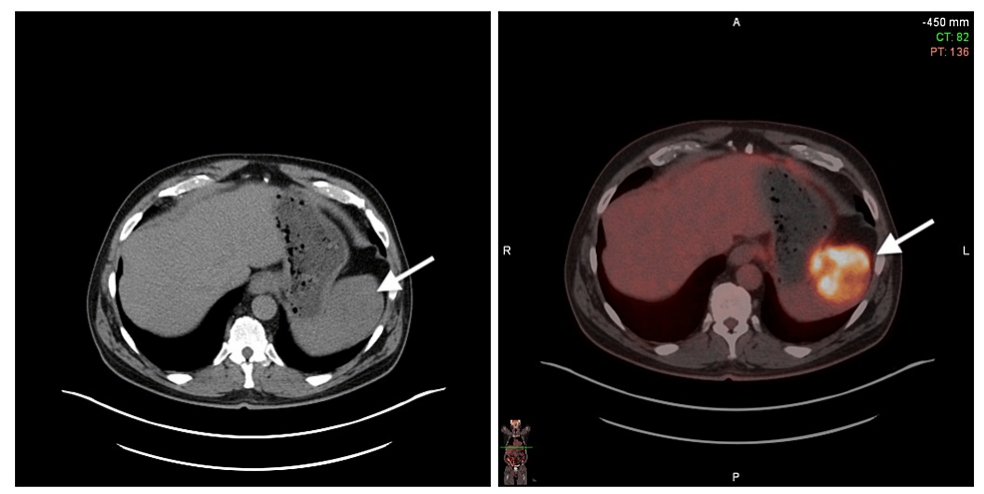
Transverse section of PET-CT scan at the level T10 showing FDG-avid splenic mass (arrow) PET-CT, positron emission tomography-computed tomography; FDG, fluorodeoxyglucose

**Figure 2:**
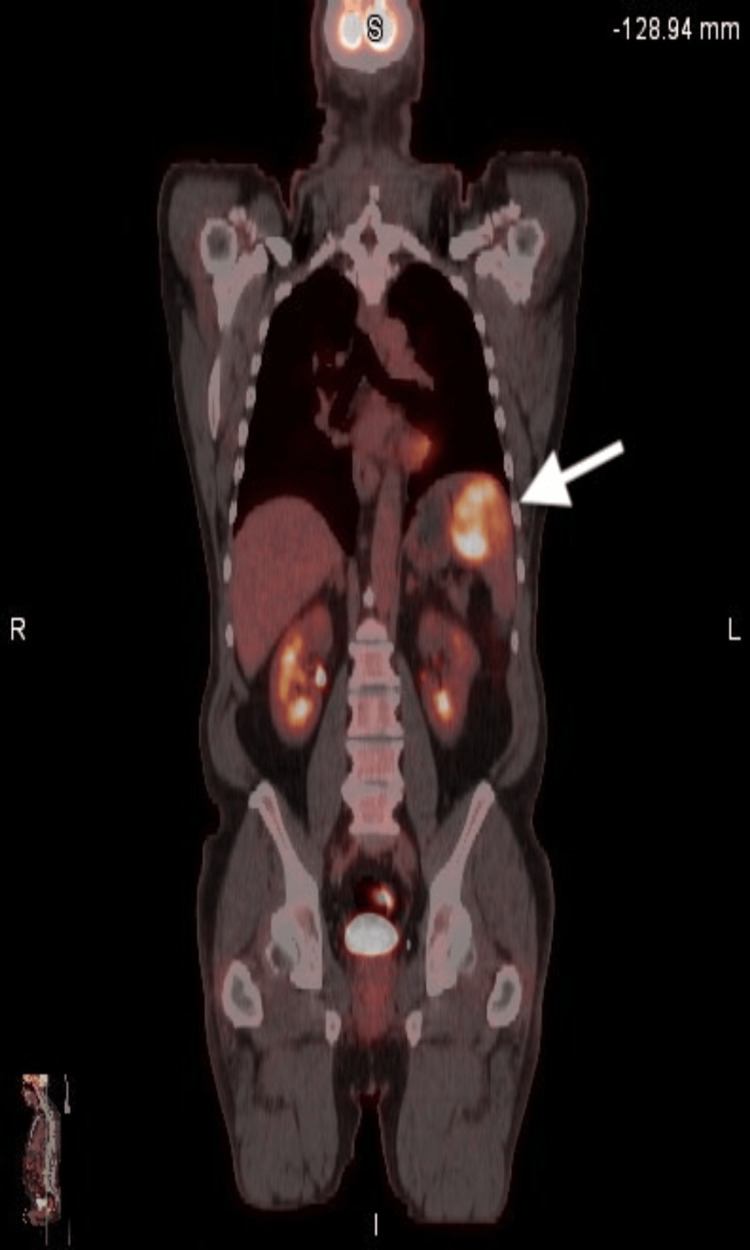
Coronal section of PET-CT scan showing solitary FDG-avid splenic mass (arrow) PET-CT, positron emission tomography-computed tomography; FDG, fluorodeoxyglucose

**Figure 3:**
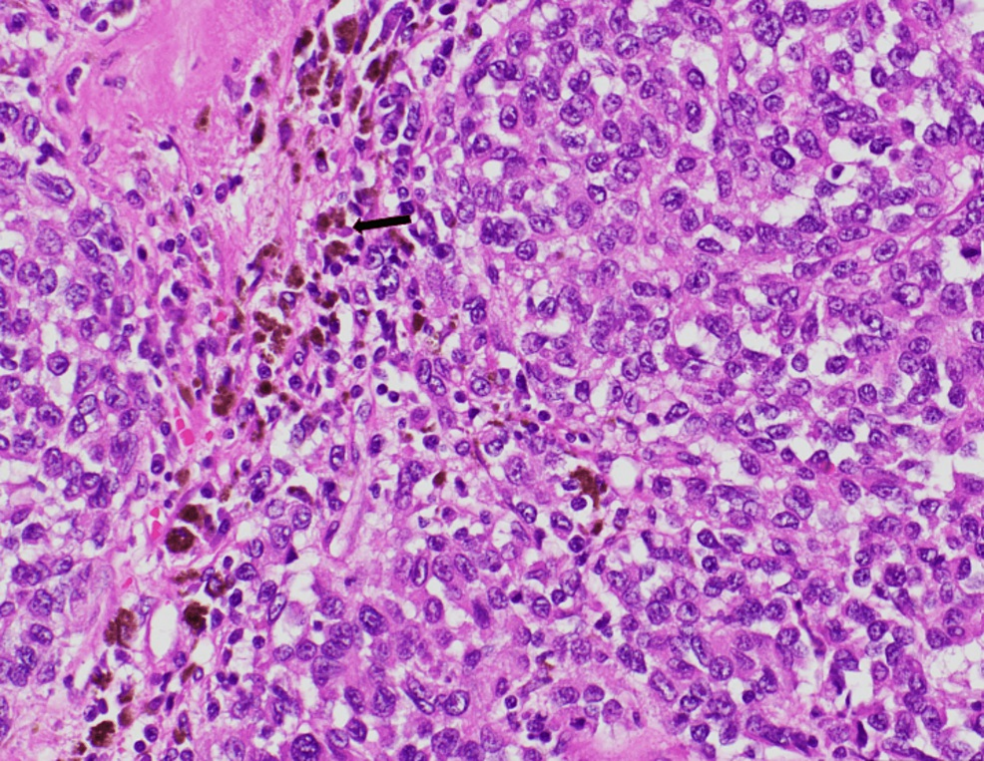
Histopathologic examination of splenectomy specimen showing pigmented melanoma cells (arrow)

PET-CT revealed that the concerned pulmonary nodule was not FDG-avid. However, there was a 6 × 6.3 × 6.6 cm solid mass in the upper pole of the spleen with intense FDG uptake of standardized uptake value (SUV) of 8.0 (Figure 1 and Figure 2). The differential diagnosis for his incidentally detected FDG-avid splenic mass with significant weight loss was thought to be lymphoma or metastasis from solid cancer. His PET-CT showed no other hypermetabolic focus in solid organs or lymph nodes. He underwent a diagnostic total splenectomy. Intraoperative findings revealed a 15 cm in length enlarged spleen with a large central hemorrhagic mass. Histology showed the presence of melanocytes and melanin deposition in splenic tissue with nuclear atypia and minimal differentiation (Figure 3). Immunochemistry was positive for SOX10, MART-1, and S100. Genomic testing for BRAF V600E mutation was negative. Pathology findings with supporting evidence from immunochemistry confirmed the diagnosis of metastatic melanoma.

Complete ocular and dermatological inspections were unremarkable for dysplastic nevus or suspicious pigmentation. Oral examination and colonoscopy did not reveal concerns for mucosal melanomas. Magnetic resonance imaging of the brain was negative for central nervous system (CNS) involvement. Our patient received postsplenectomy vaccinations and was started on adjuvant immunotherapy with a combination of nivolumab and ipilimumab for four cycles, followed by a surveillance PET-CT scan, which was negative for active disease. He has been compliant on nivolumab maintenance, and regular surveillance PET-CT scans every 3-6 months showed stable pulmonary nodule with no sign of melanoma recurrence.

## Discussion

Melanomas (also malignant melanoma) are skin cancers with challenging clinical diagnoses, as it is estimated that only two-thirds of early melanomas are identified on skin examination, even by seasoned dermatologists. However, following histopathologic confirmation, cutaneous or mucosal melanomas are classified as per 2018 World Health Organization (WHO) guidelines involving genomic analysis. V600 mutation is a gain-of-function mutation in the *BRAF* gene and was the first mutation in melanoma to be targeted for treatment [4]. Unfortunately, our patient could not be classified by the above guidelines due to the absence of cutaneous or mucosal lesions.

Although 96%-97% of melanomas have a known primary site, such as the skin, mucosa, or eye, a small subset presents with regional or distant metastasis without a primary site [5]. There has been a vague understanding of this variation ranging from the migration of metastatic melanoma cells along the neural crest to distant organs to the possibility of spontaneous resolution of the cutaneous lesion by the host’s immune system following metastasis [6]. A retrospective study of 2485 melanoma patients showed the incidence of unknown primary to be 2.6%, of which 43% had lymph node metastasis and 38% had visceral involvement (40% had CNS involvement). Patients with regional metastasis to lymph nodes had a better five-year survival rate than visceral metastasis. There was no report of splenic involvement in this study [7].

The spleen is a relatively uncommon site for metastasis due to its anatomy and the inhibitory effect of the splenic microenvironment on the metastatic cells. Splenic metastases occur in the context of multi-visceral disseminated cancer. Tumors that usually metastasize to the spleen include melanoma and tumors of the pancreas, breast, lung, and ovary [2]. Several studies have shown that an isolated splenic mass, as seen in our patient, is an uncommon initial presentation of metastatic melanoma [8,9]. As per the American Joint Committee on Cancer (AJCC) eighth edition melanoma staging, which includes whole-body imaging and LDH levels, any visceral organ involvement is under the purview of metastatic disease [10].

Identifying BRAF V600E mutation in cancer cells guides treatment management. Without this mutation, combination immunotherapy with ipilimumab and nivolumab followed by nivolumab maintenance is the preferred treatment option in eligible patients. These recommendations stem from a phase III landmark CheckMate 067 trial showing improvement in objective response rates with a combination of ipilimumab and nivolumab compared to either agent alone in patients with treatment-naïve advanced melanoma [11]. The management of distant resectable metastasis would involve surgery with adjuvant immunotherapy or targeted therapy if BRAF mutated. Although the prognosis of melanoma patients with splenic metastasis is generally poor, splenectomy has improved the quality of life and long-term disease-free survival of patients in good clinical condition [3,12]. Our patient with isolated splenic metastasis of BRAF-negative melanoma underwent therapeutic total splenectomy with adjuvant ipilimumab and nivolumab immunotherapy.

## Conclusions

Solitary splenic metastasis of melanoma is a rare presentation. It has been described in the context of known primary melanoma. It is rarely reported without primary cutaneous or ocular melanoma. Based on our literature review, only two case reports have been published before this. Metastatic melanoma of the spleen without cutaneous or ocular melanoma is usually asymptomatic and detected at the time of autopsy. However, with the widespread use of PET-CT, these presentations are being diagnosed earlier. It should be considered in differential along with lymphoma and splenic abscesses while evaluating FDG-avid splenic mass. Total splenectomy can be diagnostic and therapeutic for isolated FDG-avid splenic masses. In addition to splenectomy, metastatic melanoma of the spleen is managed with adjuvant immunotherapy or targeted therapy.
